# Effects of transport time and feeding type on weight loss, meat quality and behavior of broilers

**DOI:** 10.5713/ab.21.0381

**Published:** 2022-01-21

**Authors:** Yajie Fu, Jingwen Yin, Ning Zhao, Ge Xue, Runxiang Zhang, Jianhong Li, Jun Bao

**Affiliations:** 1 College of Life Science, Northeast Agricultural University, Harbin, Heilongjiang 150030, China; 2 College of Animal Science and Technology, Northeast Agricultural University, Harbin, Heilongjiang 150030, China

**Keywords:** Arbor Acres Broiler, Feeding Methods, Meat Quality, Stress Behavior, Transport Time, Weight Loss

## Abstract

**Objective:**

The purpose of this study is to determine the optimal time of transportation of floor-feed and scatter-feed broilers.

**Methods:**

Eighty healthy Arbor Acres (AA) broilers (21-day-old, 624.4 g, male, standard error = 6.65) were selected and randomly divided into two experimental groups (floor-feed and scatter-feed), then fed for three weeks. The experiment comprised a 2×4 factorial design with 2 feed patterns (floor-feed and scatter-feed) and 4 transport periods (2, 3, 4, and 5 h), and 4 replicates of 5 broilers (54-day-old, 2243 g, standard error = 46.65) was used to compare weight loss, meat quality and behavior index of different groups.

**Results:**

It appeared that drip loss, meat color and resting behavior of experimental broilers changed as length of transportation (p<0.05), however, weight loss and pH were not significantly transformed (p>0.05). Compared with floor-feed group, broilers in scatter-feed group had lower pH at 24 hours (3 h) and different behavioral indicators (p<0.05). Especially indicators after 3 h transportation, there were obvious differences between the two feeding modes in the behavior reaction of stress events before slaughter with different transport duration (p<0.05). The fluctuation of data on resting behavior with scatter-feed was significantly higher than that of floor-feed broilers. There was no interaction between transport time and different feeding methods for index tested of our experiment (p>0.05).

**Conclusion:**

Comprehensive analysis showed that the maximum transport duration of floor-feed and scatter-feed broilers should not exceed 3 h, and scatter-feed broilers were more likely prone to fear.

## INTRODUCTION

Consumers prefer scientifically raised broiler meats rather than other meats because of their low-price and easy-to-cook properties. Whereas, to expand poultry production blindly would produce more frequent transport impairments such as welfare reduction, weight loss, and meat metamorphism [1–3]. Therefore, to pursue efficient economic returns, transport stress must be reduced in the scientific and rapid production of broilers.

Many techniques to decrease the transport stress of birds have been used, such as feed addition [4,5], improving transportation and vehicle facilities [6,7], microclimate temperature control [8], etc. Compared to these methods, controlling transport distance is the most economical way to decrease transport stress. Considering with the increased scale of industrial intensive poultry farming, reasonable thresholds for transport distance should be determined to ensure the meat quality and welfare of commercial broilers before slaughter.

As an important premortem stressor, the influence of transport stress does not stop at slaughter, but also continues to affect conversion of muscle to meat [9,10]. Different transport times could alter stress levels by changing oxidation levels, heat production, and pH, which then manifest as altering cytochrome content, protein content and configuration, and internal and external water distribution [10,11]. The damaged appearance of raw meat with too dark or too light color and low water-holding ability may reduce purchasing desire and eating experience of consumers [12,13]. Because of higher antioxidant levels with higher muscle fiber strength, mitochondrial function, nutrient content and lower glycogen content, meat quality of free-range broilers seems fresher than plain broilers [14]. Floor-feed is generally adopted in commercial poultry farming. Studies have found that mortality and weightlessness of broilers increase with the extension of transportation time [1,2]. The results of floor-feed broilers in the experimental base were not the same. Owens and Sams [9] found that the pH of pectoral muscle increased significantly and L* decreased significantly after transportation for 3 h. Gou et al [3] found that antioxidant capacity and body weight of broilers decreased, but there was no significant difference in meat quality including pH and meat color. Pan et al [6] found in the study that the pH of floor-feed broilers under experimental conditions after 3 h transportation did not change significantly, a* significantly decreased, b* significantly increased, and the deterioration of meat quality was significantly decreased after the addition of antioxidant. Free-range farming can change meat quality and body performance of poultry [14]. It was found that plasma corticosterone content of two organic scatter-feed hens decreased rapidly during the first 6 hours of transportation [15]. However, there is still a lack of clear comparison results about the effects on floor-feed and scatter-feed broilers after transportation time.

As recognized by the World Organization for Animal Health (OIE) good animal welfare during transport is one of the most important pre-harvest variables with respect to meat quality and should be considered a critical control point [16, 17]. Behavioral changes reflect emotional changes caused by transport stress. Cashmana et al [18] found that the degree of stress fluctuated with prolonged transport stress before slaughter. Due to differences in broiler breeds and growing environments, there are differences in the psychological and physiological sensitivities of organisms to transport pressure [19]. We hypothesize that some effect would be caused by transport time on broilers. After a certain period of transportation, the damage of stress exceeds the homeostatic adjustment and recovery ability of broilers, leading to significant increase in weight loss and deterioration of meat quality. At the same time, the degree of fear in broilers may be difficult to alleviate and will produce obvious changes in behavior indicators. However, there may be differences between broilers reared in floor-feed and scatter-feed.

The objective of the present study was to determine the appropriate transportation time for floor-feed and scatter-feed broilers by testing the effects of the different transportation time on weight loss, meat quality and behavior in broilers.

## MATERIALS AND METHODS

### Experimental animals and design

All experimental protocols and procedures were approved by Northeast Agricultural University Animal Care and Use Committee (IACUCNEAU20150616).

Arbor Acres broilers (AA) (n = 100, male, 1-day-old) with 39.59 g of average initial body weight (standard error = 2.09) were selected as test animals. During the first 21 days, the floor of AA broilers was padded with 10 cm thick sawdust, with a feeding density of 8 to 10 birds/m^2^. Natural ventilation with 60% to 70% of relative humidity was used in the chicken house. The temperature inside (34°C to 35°C in the first 7 days, reduced by 2°C to 3°C every 7 days until 18°C to 20°C) and ordinary incandescent lighting (23 h in the first 3 days; then 12 h every day, from 7:00 am to 7:00 pm) were strictly controlled. Three-week-old broilers (n = 80) with 624.4 g (standard error = 6.65) were randomly selected and divided into two groups, including floor-feed and scatter-feed groups. The floor-feed group (n = 40) was raised on the indoor floor (relative humidity, 48% to 50%; stable temperature, 18°C to 20°C; feeding density, 8 birds/m2, natural light). Scatter-feed group (n = 40) was raised in a plastic greenhouse with a metal frame covered by the plastic film (4 m length, 2 m width, 1.2 m height, 5 cm thick padding replaced every 4 days; relative humidity, 30% to 40%; stable temperature, 22°C to 26°C; feeding density, 5 birds/m^2^) as a sports field (activities and rest with sun-shade net) during day-time (from 7.00 am to 5.00 pm), and was forced into chicken house during night-time. Experimental broilers were given free access to feed and water. Broilers were fed with an early-stage diet (metabolizable energy of 12.1 MJ/kg and crude protein of 21.0% for 1 to 21 days of age) and a late-stage diet (metabolizable energy of 12.6 MJ/kg and crude protein of 19.0% for 22 to 43 days of age).

The forty 54-day-old healthy broilers (2,243±46.65 g) from each of the above two feeding methods were weighted and randomly divided into four groups (4 replicates and 5 chickens each). After 10 h of limited feeding treatment, all chickens were caged and transported (5 per carton). Each group of broilers was transported between the country roads for 2, 3, 4, and 5 h, on the day of transportation, the weather was overcast, and the average temperature was 20°C. The broilers from each treatment group were immediately weighted, then rested for 1 hour, hung for 12 seconds, and killed by decapitation. During the resting and hanging period, a video camera, GoPro2 HD (GoPro Inc., San Mateo, CA, USA) was used to record the behavior of chickens. The pectoral muscle of broiler chickens was taken and examined.

### Sample collection and index determination

#### Weight loss


$$\text{Weight loss }(\text{Ws})=\text{body weight before fasting }({\text{W}}_{1})-\text{body weight before slaughter }({\text{W}}_{2})$$

#### Meat quality

##### i) pH1 and pH_2_

Acidimeter was calibrated before the sample determination using a standard solution of pH (4.003, 6.864, and 9.182). pH_1_ and pH_2_ of breast muscles were determined by a pH meter within 45 to 60 min and 24 hours, respectively (Elta-320; Mettler Toled, Shanghai, China). The probe was inserted directly into muscle tissue, and the reading was acquired after stabilizing it. The breast muscles were wrapped with plastic wraps and stored at 4°C after determining pH.

##### ii) Meat color

The breast muscle was cut, and the thickness of the muscle was 1.5 cm. The meat color (L*, a*, and b* values) was determined using a Chroma meter (CR-400; Konica Minolta, Tokyo, Japan) at 1 h after the slaughter in three regions of the left pectoral muscle. Finally, the average value was obtained.

##### iii) Determination of drip loss

After slaughter, breast muscles were trimmed into meat cuts (5 cm×3 cm×3 cm), and the weight of breast muscles (W_1_) was measured. Then, the meat cuts were tied with thin wires and transferred to a 50 mL cold storage tube at 4°C for 24 h. After absorbing surface moisture using a clean filter paper, meat cuts were reweighed (W_2_). Drip loss percentage was calculated according to the formula: Drip loss (%) = (W_1_–W_2_)/W_1_×100%.

##### iv) Shear force

After determining drip loss, meat samples were placed in food bags and then placed in a water bath pot at 80°C until the center temperature of meat reached 75°C and maintained at 75°C for 10 min. Then, meat samples were cut into 2.5 cm and 1.0 cm diameters using scissors and sheared using a tenderometer (C-LM3; College of Engineering, Northeast Agricultural University, Harbin, China) for the maximum shear force. The Warner-Bratzler single blade sheared the meat column in the direction perpendicular to the muscle fiber, with the shear velocity of 5 mm/s. Each meat sample was cut 3 times, and the average value was obtained.

#### Behavior

GoPro2 HD (GoPro Inc., USA) (resolutions: 1,080 p: 1,920, 1,080, and 30 FPS) was used to avoid interference by direct contact with the animals. The cameras were placed around the broiler lounge and the slaughter room in the same arrangement.

##### i) Behavior during rest

After the end of the transport, the videos were recorded after the broiler entered the lounge to adapt to the environment for 30 min. The recording time was 30 min. During the behavior observation period, it was sampled instantly every 30 s. The behavior data of broilers were expressed by the number of activities occurring during observation time. Behavior definitions are shown in Table 1.

##### ii) Struggling behavior before slaughter

The total duration of wing flapping was recorded from hanging to slaughter. Vocalisations from the beginning of poultry hanging observation were scored according to the corresponding four levels: a) poultry does not sound, score 0; b) poultry makes a simple weak sound, score 1; c) poultry makes a weak sound for a long time, score 2; d) poultry makes a bright sound for a long time, score 3.

### Statistical analysis

The test data of weight loss, meat quality and behavioral indicators were tested by the Univariate process in the SAS9.1 before the analysis. The quantitative data, which did not conform to the normal distribution, were converted by log, sine and inverse string, square root, etc. One-way analysis of variance (ANOVA) analysis was done, and the multiple comparisons were carried out by the Duncan test method.


$$\text{Experiment II:}\;{\text{y}}_{\text{ij}}=\mu {\text{E}}_{\text{i}}\;{\text{W}}_{\text{j}}\;{\left(\text{WE}\right)}_{\text{ij}}{\text{e}}_{\text{ij}}$$

μ, total mean effect; E, feeding methods; W, transport time; e, random error.

## RESULTS

### Weight loss

As shown in Table 2, transport time had no significant effect on weight loss both in the floor-feed and scatter-feed broilers (p>0.05).

### Meat quality

Table 3 shows that the impact of the transport phase on floor-feed and scatter-feed broilers’ meat quality is multifaceted. Disparate transport time had no effect on pH_1_ and pH_2_ (p>0.05), but resulted in significantly increased drip loss, flesh-color L* both in floor-feed and scatter-feed groups (p< 0.05), while reduced a* value only with scatter-feed (p<0.05). The meat quality comparison on transportation revealed a higher pH_2_ (3 h) in floor-feed than that in scatter-feed group (p<0.05). There was no significant interaction between feeding mode and fasting length of all broiler indicators for the data of meat quality in our experiment (p>0.05).

### Behavior of broilers during rest

As shown in Table 4, the changes in behavior at rest differed according to time spent in transport, with remarkable reduction in up-regulation energy-consuming behavior (walking in 4, 5 h; standing in 3, 4, 5 h; p<0.05), conversely substantial increase in reduced energy-consuming behavior (lying in 3, 4, 5 h; p<0.05) in scatter-feed broilers. Fluctuation in other natural behavior also was associated with transport duration with scatter-feed: lower preening in 4 h (p<0.05); less head moving in 5 h (p<0.05); diminished meaningless pecking in 3 h and 5 h (p<0.05); nevertheless, no remarkably changed foraging (p>0.05). All behavior of floor-feed broilers during rest was shown with no significant alteration except for lying which was higher after 3, 4, and 5 h transport than in 2 h group (p<0.05).

When the behavior indicators of each group were collected, all the behavior during rest was different between the feeding methods (p<0.05). And most of the data showed the two feed patterns significantly differed after 3 h transport (p<0.05) in Table 4, while at 4 h, there was no obvious change in all behavioral indicators (p>0.05). Standing, walking, and preening, foraging behavior of floor-feed broilers were significantly lower than that with scatter-feed at 3 h, while lying and head movement behavior were significantly higher (p<0.05).

### Struggling behavior before slaughter

As shown in Table 5, transportation time and feeding mode have no significant influence on the pre-mortem struggle behavior of broilers (p>0.05).

## DISCUSSION

### Effects of different transport times on weight loss of broilers

Transport stress results in serious weight loss, increasing injury rates, reducing production performance, and causing mass deaths in severe cases [1–3]. Weight loss occurred due to energy consumption and excretion, during poultry transportation. Compared with some studies that showed a huge difference in weightlessness [3,20], there was no prominent effect of transport time on the weight loss of poultry in our study, which is mainly due to the small number of broilers and better feeding conditions. At the same time, we carried out a unified long-term feeding treatment for broilers before transportation and reduced the weight effect caused by different excreta exclusion types in different feeding methods.

### Effects of different transport times on meat quality of broilers

The appearance quality of poultry meat is a key factor which influences consumers’ purchase desire and cooking experience. Transport stress can devalue ketone bodies resulting in changing flesh color and increasing drip loss [21]. Conspicuous changes in drip loss, a* value and L* value were found, suggesting that prolonged transport stress time reduced meat quality.

The main factors effecting the color of poultry meat are the content of myoglobin and the chemical state and reaction of myoglobin of meat which were closely impacted by the products of transport stress [22,23]. Metabolite products (such as reactive oxygen species, free radicals, lactic acid or phosphoric acid) can damage sensitive muscle cell membranes, resulting in myoglobin infiltration into the blood and an increase in L* [4,11,21]. The content of myoglobin in breast muscle cells is low, and pale, soft and exudative (PSE) meat is easily stimulated by pre-mortem stress [7,21]. The L* value was significantly increased with transportation time as noted in our experimental results (p<0.05), and the value in the 4 h group began to be higher than 50, suggesting the risk of negative meat quality change as a L* value above 50 increases the risk of PSE meat [21]. The red value (a*) of meat is usually influenced by pigment state and structure [21,23]. The fresh muscle colour favoured by consumers mainly comes from the red oxygenated myoglobin which can reversibly convert to the brown metmyoglobin and is affected by the changing temperature, pH, oxidation degree of the transport pressure [11,21,23]. In this experiment, a* value of scatter-feed broilers changed significantly with the extension of transportation time. In view of the insignificant changes in muscle pH, we believe that it is mainly due to the cellular oxidative stress caused by the emotional stress. The level of fear was increased when scatter-feed broilers were moved from free rearing space to narrow cages. The product of cellular oxidative stress alters the state of hemoglobin, changing the red color of muscle [21]. Our experimental results were the same as Pan et al [6] and the pectoral muscle a* decreased significantly at 3 h. As the muscle of white meat, consumers are not sensitive to small amounts of light red. The influence of meat color on purchasing desire mainly depends on L* value.

The water loss of meat quality was affected by the hydrophilic ability of intracellular proteins and the integrity of the cell structure [11,21]. Transport stress cause drip loss because free radicals and acidic substances produced after transport will undermine the protein structure and decrease its hydrophilicity, damaging the cell membrane and making the exosmosis of cells contents [21–23]. High drip loss will affect the taste of raw meat when it is converted to cooked meat [22,23]. The mean values of different feeding groups and the two groups in the 4 h group were significantly increased, suggesting that the threshold of cell self-regulation may have been exceeded.

Contrary to the prediction, transport stress did not cause a significant change in pH_1_ which is affected by the level of glycolysis in cells. This is caused by the removal of acid substances during the recovery process, and the irreversible damage to the cell structure cannot be recovered immediately, so that meat quality does not change synchronously. Therefore short-distance transport stress increases mortality, while prolonged rest reduces meat injury [1,22]. Of course, it has been suggested that economic losses caused by ketone body depreciation can be reduced by extending the rest period before slaughter, but this seems inconsistent with the purpose of efficient intensive production [22].

The effect of feeding methods on pH value is different from that of Dal Bosco et al [24], the pH_2_ of scatter-feed broilers at 3 h was significantly lower than that of our different results. pH_2_ which negatively correlated with glycogen content was different from pH_1_ which mainly affected by the level of cell glycolysis [21,24]. On the one hand, our breeding space was not big enough for poultry to fly much, so as the glycogen content in muscles was not low before transportation. On the other hand, the activity resting behavior of floor-feed broilers during the recovery process was significantly higher than that of scatter-feed broilers (p<0.05), which increased the consumption of glycogen reserve. Finally, the level of pH_2_ of scatter-feed broilers in 3 h group was lower than that in floor-feed group.

### Effects of different transport times on behavior during rest of broilers

Behavior is an important indicator of animal welfare. In a naturally stress-free environment, birds could show natural behavior, such as walking, exploring, foraging, fighting, decorating, and so on. When the poultry is in a state of stress, it produces behavioral disorders. Poultry behavior changed from excitement and anxiety to inhibition and depression with prolonged stress source stimulation time [18]. Dalton pointed that long-term transport pressure increased the attacking behavior of fire chickens [25]. Cashmana et al [18] also found that the tension static immobility time of chickens increased significantly, and then chickens were in fear stress. Our study showed that, the activity behavior decreased as longer transportation, suggesting that the anxiety of birds increased, which is consistent with Cheng and Jefferson [26]. But behavioral indicators in our study shown that, the stress response intensity was not simply negatively correlated with the length of transport time. Poultry behavior activity increased in the acute stress period to relieve the transport stress, while the chronic stress period decreased suggesting that poultry welfare has been compromised beyond broilers’ self-regulating ability. As a whole, 3 h transport treatment seems to be the tipping point for behavioral change. In addition, as transport time increases, behaviour that could increase energy expenditure (standing and walking) are negatively correlated with behaviour that could decrease energy expenditure (lying), which can maintain stable cellular glycogen levels and change not significantly in pH_2_ [27].

Behavioral differences may also depend on different tissue function with floor-feed and scatter-feed. Due to the limited growth space and insufficient leg training, the bones of floor-feed group could not be effectively strengthened, and the mitochondrial activity of muscle cells was low [19]. Similar to our study, walking and standing behavior were least observed in floor-feed broilers in previous studies [28,29].

### Effects of different transport times on struggling behavior before slaughter of broilers

Poultry generally reacts to hanging by struggling, winging, screaming, or trying to raise their heads. Longer flapping time in hanging behavior can be regarded as an indication of escape behavior and discomfort [30]. The long flapping of wings indicates that poultry has a great sense of physical stress and psychological fear. Loud and long-term calls are natural responses of poultry to acute stress. Our results showed that the transport time had no significant effect on the flapping behavior and vocalization behavior of broilers before slaughter, which may be due to the panic and psychological stresses caused by the transport on poultry being relieved after resting for one hour at the end of the transport. Other acute stress factors concealed the effects of transport stress on poultry during the observation of the hanging experiment, and no significant differences were obtained. The experiment of M Debut was inconsistent with our findings and show that hanging influenced pre-mortem struggling behavior of poultry [31], which was due to the effect of hanging on pre-mortem struggling behavior of poultry without considering the factors of transport stress. And similarly, as Debut, our study showed that the pH_2_ of breast muscles changes synchronously with preslaughter struggle behavior. Again, the data suggests that glycogen level of breast muscle was changed by behavioral intensity after transport which would ultimately change pH_2_ level.

Transportation is inevitable in the process of poultry production. The transport time is affected by the distance from the farm to the slaughterhouse, the speed of the vehicle, choice of the route of transportation and weather conditions. As our experimental result reveals, the planning of comprehensive production and processing enterprises and the establishment of unified safety standards for transportation, transport time and feeding methods are important standards to be considered.

## CONCUSION

In summary, the transport time threshold for broilers is 3 hours. Over 3 hours of transportation, broilers cannot alleviate the damage caused by transportation stress, resulting in lower L* value of meat color and excessive drip loss. Scatter-feed broilers are more susceptible to panic than floor-feed broilers, which requires attention from the breeder during transportation to ensure the welfare of broilers.

## Figures and Tables

**Table 1:** Behavioural categories and their definitions

Items	Definition
Behavioral variables	
Head movement	The head of the poultry rotates rapidly, indicating that it is visually alert to the surroundings
Preening	The beak is used to comb through or manipulate any area of feathers on the bird’s own body
Meaningless pecking behavior	Non-offensive peck on other things, including a cage, a feather, an empty peck
Foraging	Peck the floor while planing or head moving below the tail
State behavior	
Walking	At least one step or more
Standing	Legs landing without showing other state behavior in the definition
Lying	Bird is in the sitting position, motionless with its body contacting the floor of the crate

Debut et al [31].

**Table 2:** Effect of the length of transport and the feed pattern on the fresh weight of broilers at 54-days

Items	Feeding pattern	Length of transport (h)^1)^					P_L_	Effect^2)^		
	
		0	2	3	4	5		Pattern	Length	Pattern ×length
Weight (g)	Floor-feed	2,249.58±42.15	2,140.21±150.23	2,155.07±32.14	2,113.95±45.45	2,116.67±41.89	NS	NS	NS	NS
	Scatter-feed	2,231.43±35.67	2,143.17±26.35	2,109.16±37.41	2,156.32±32.63	2,026.51±97.43	NS			
	P_F_	NS	NS	NS	NS	NS				
Weight loss (g)	Floor-feed	-	104.00±108.51	93.00±20.22	122.22±34.84	124.00±29.72	NS	NS	NS	NS
	Scatter-feed	-	80.00±6.61	112.00±14.02	95.00±11.93	235.00±109.82	NS			
	P_F_	-	NS	NS	NS	NS				

P_L_, the effect of transportation time on fresh weight; P_F_, the effect of feeding pattern on fresh weight.

1) Results of one-factor analysis of variance.

2) Results of two-factor analysis of variance.

NS, no significant difference (p>0.05).

**Table 3:** Effect of the length of transport and the feed pattern on breast meat quality of broilers at 54-days

Items	Feeding pattern	Length of transport (h)^1)^				P_L_	Effect^2)^		
	
		2	3	4	5		Pattern	Length	Pattern ×length
pH_1_	Floor-feed	6.82±0.13	6.65±0.10	6.88±0.07	6.80±0.07	NS	NS	NS	NS
	Scatter-feed	6.53±0.14	6.80±0.11	6.63±0.12	6.61±0.10	NS			
	P_F_	NS	NS	NS	NS				
pH_2_	Floor-feed	6.35±0.06	6.35^x^±0.04	6.29±0.05	6.41±0.08	NS	NS	NS	NS
	Scatter-feed	6.31±0.14	6.21^y^±0.04	6.25±0.23	6.39±0.04	NS			
	P_F_	NS	0.037	NS	NS				
Drip loss	Floor-feed	1.94^b^±0.20	3.44^ab^±1.07	4.27^a^±0.87	4.64^a^±0.68	0.024	NS	0.021	NS
	Scatter-feed	2.13^b^±0.22	4.52^ab^±1.46	5.10^a^±1.91	5.34^a^±1.58	0.037			
	P^F^	NS	NS	NS	NS				
Shearing force	Floor-feed	2.66±0.51	3.15±0.26	2.45±0.53	2.28±0.58	NS	NS	NS	NS
	Scatter-feed	2.78±0.44	2.38±0.60	1.09±0.57	2.04±0.47	NS			
	P_F_	NS	NS	NS	NS				
L^*^	Floor-feed	45.32^b^±1.09	46.53^ab^±1.88	50.75^a^±1.98	50.51^a^±1.23	0.031	NS	0.014	NS
	Scatter-feed	45.57^b^±1.22	47.32^ab^±1.03	49.79^ab^±2.57	50.94^a^±1.23	0.024			
	P_F_	NS	NS	NS	NS				
a^*^	Floor-feed	0.36^y^±0.13	0.78±0.34	1.05±0.50	1.11±0.21	NS	NS	NS	NS
	Scatter-feed	1.45^ax^±0.25	0.47^b^±0.24	0.85^ab^±0.18	1.07^ab^±0.23	0.03			
	P_F_	0.046	NS	NS	NS				
b^*^	Floor-feed	12.39±0.45	12.46±0.37	12.05±0.41	11.56±1.31	NS	NS	NS	NS
	Scatter-feed	12.76±0.86	11.69±1.00	10.84±1.00	11.39±1.29	NS			
	P_F_	NS	NS	NS	NS				

P_L_, the effect of transportation time on breast meat quality; P_F_, the effect of feeding pattern on breast meat quality.

1) Results of one-factor analysis of variance.

2) Results of two-factor analysis of variance.

NS, no significant difference (p>0.05).

a,b Significant difference in a row (p<0.05).

x,y Significant difference in a column (p<0.05).

**Table 4:** Effect of the length of transport and the feed pattern on behavior during rest of broilers at 54-days

Items	Feeding pattern	Length of transport (h)^1)^				P_L_	Effect^2)^		
	
		2	3	4	5		Pattern	Length	Pattern ×length
Standing	Floor-feed	12.33^y^±3.06	6.33^y^±1.62	6.67±3.80	4.00^y^±1.13	0.037	0.016	0.009	NS
	Scatter-feed	51.67^ax^±5.75	22.67^bx^±2.34	17.33^b^±5.42	26.67^bx^±3.21	0.029			
	P_F_	0.020	0.016	NS	0.024				
Walking	Floor-feed	2.00±0.82	1.33^y^±0.62	0.67±0.41	0.67±0.67	NS	0.033	0.027	NS
	Scatter-feed	3.63^ab^±1.70	7.67^ax^±2.01	1.67^b^±1.67	2.33^b^±0.67	0.023			
	P_F_	NS	0.024	NS	NS				
Lying	Floor-feed	85.67^bx^±3.36	92.33^abx^±2.07	92.67^ab^±4.10	95.33^ax^±1.22	0.044	0.015	0.007	NS
	Scatter-feed	44.67^by^±6.78	69.67^ay^±3.05	81.00^a^±7.04	71.00^ay^±3.44	0.032			
	P_F_	0.039	0.011	NS	0.021				
Head movement	Floor-feed	5.60±0.98	6.40^x^±0.68	5.60±2.93	3.20±1.50	NS	NS	0.042	NS
	Scatter-feed	4.40±0.51	3.60^y^±0.87	2.60±0.87	2.80±0.80	NS			
	P_F_	NS	0.012	NS	NS				
Preening	Floor-feed	0.40^y^±0.25	0.20^y^±0.20	0.60±0.60	0.60±0.60	NS	0.031	NS	NS
	Scatter-feed	2.80^ax^±0.37	1.40^abx^±0.40	0.60^b^±0.25	2.40^a^±0.87	0.033			
	P_F_	0.009	0.037	NS	NS				
Foraging	Floor-feed	0.80±0.37	0^y^	0.40±0.40	0^y^	NS	0.028	NS	NS
	Scatter-feed	4.20±2.33	8.40^x^±1.21	4.20±2.96	5.00^x^±1.76	NS			
	P_F_	NS	<0.001	NS	<0.001				
Meaning-less pecking	Floor-feed	0.20^y^±0.20	2.00±1.52	2.00±0.71	3.00±1.55	NS	0.019	NS	NS
	Scatter-feed	8.00^ax^±1.93	1.00^b^±0.32	4.20^ab^±1.40	1.60^b^±0.75	0.036			
	P_F_	0.033	NS	NS	NS				

P_L_, the effect of transportation time on behavior during rest; P_F_, the effect of feeding pattern on behavior during rest.

1) Results of one-factor analysis of variance.

2) Results of two-factor analysis of variance.

NS, No significant difference (p>0.05).

a,b Significant difference in a row (p<0.05).

x,y Significant difference in a column (p<0.05).

**Table 5:** Effect of the length of transport and the feed pattern on struggle behavior before slaughter of broilers at 54-days

Items	Feeding pattern	Length of transport (h)^1)^				P_L_	Effect^2)^		
	
		2	3	4	5		Pattern	Length	Pattern ×length
Vocalisations	Floor-feed	2.60±0.51	3.20±0.09	2.80±0.58	2.40±0.40	NS	NS	NS	NS
	Scatter-feed	3.40±0.40	3.60±0.40	3.60±0.40	3.40±0.40	NS			
	P_F_	NS	NS	NS	NS				
Total duration of wing flapping	Floor-feed	5.60±1.47	5.20±1.66	4.80±0.37	4.40±1.22	NS	NS	NS	NS
	Scatter-feed	5.80±1.02	4.80±1.24	4.20±1.11	2.40±1.29	NS			
	P_F_	NS	NS	NS	NS				

P_L_, the effect of transportation time on struggle behavior before slaughter; P_F_, the effect of feeding pattern on struggle behavior before slaughter.

1) Results of one-factor analysis of variance.

2) Results of two-factor analysis of variance.

NS, no significant difference (p>0.05).
